# A Review of COVID-19 Response Challenges in Ethiopia

**DOI:** 10.3390/ijerph191711070

**Published:** 2022-09-04

**Authors:** Abdulnasir Abagero, Luca Ragazzoni, Ives Hubloue, Francesco Barone-Adesi, Hamdi Lamine, Adamu Addissie, Francesco Della Corte, Martina Valente

**Affiliations:** 1 CRIMEDIM—Center for Research and Training in Disaster Medicine, Humanitarian Aid and Global Health, Università del Piemonte Orientale, 28100 Novara, Italy; 2 ReGEDiM—Research Group on Emergency Disaster Medicine, Vrije Universiteit Brussel (VUB), 1050 Brussels, Belgium; 3 Department of Preventive Medicine, School of Public Health, College of Health Sciences, Addis Ababa University, Addis Ababa 70710, Ethiopia; 4 Department of Sustainable Development and Ecological Transition, University of Eastern Piedmont, 28100 Novara, Italy; 5 Department of Translational Medicine, University of Eastern Piedmont, 28100 Novara, Italy; 6 Faculty of medicine Ibn Aljazzar of Sousse, University of Sousse, Sousse 4002, Tunisia

**Keywords:** pandemic response, resilience, COVID-19, healthcare system

## Abstract

Background: The COVID-19 pandemic has positioned fragile healthcare systems in low-income countries under pressure, leading to critical gaps in service delivery. The pandemic response demands the healthcare system to be resilient and continue provision of healthcare services. This review is aimed at describing the healthcare response challenges during the pandemic in Ethiopia. Methodology: Eligible studies dealing with challenges of the healthcare system in response to the COVID-19 pandemic in Ethiopia were included. The six World Health Organization (WHO) healthcare system building blocks were used to categorize healthcare challenges. PubMed ProQuest, databases were searched, and results were summarized using systematic review synthesis. Results: Financial constraints led to a shortage of mechanical ventilators. Furthermore, the pandemic hindered the capacity to avail full packages of personal protective equipment in health facilities and intensive care capacity. The pandemic also affected the delivery of maternal, child and new-born services, prevention, and treatment of childhood illness, including immunization services. Conclusions: The COVID-19 pandemic posed various challenges to the performance of the healthcare system in Ethiopia. It is recommended that policy makers and stakeholders enhance pandemic preparedness and strengthen response capacity by considering the six WHO healthcare system building blocks.

## 1. Introduction

The pandemic response demands a healthcare system to be resilient, meaning to be able to bounce back and continue providing services in times of crisis. Nevertheless, the COVID-19 pandemic negatively impacted healthcare systems in low-income countries, highlighting critical gaps. This ultimately affects global health security [1,2]. In low- and middle-income countries, the inadequate access to healthcare services, poverty, high prevalence of comorbid diseases, and inadequate access to clean water, were exacerbated by the arrival of the COVID-19 [3]. In fact, the pandemic increased demands for intensive care, while hospital bed capacity, ventilators, and healthcare resources supplies, such as oxygen, were limited [4]. The imposition of restrictions measures to counteract the spread of the COVID-19 resulted in disruption access to healthcare services. The pandemic also resulted in increased costs of healthcare services and exacerbated reluctance to seek them due to fear of infection [5]. 

As part of the response measures, African health ministers endorsed the Joint Continental Strategy for COVID-19 on the 22 February 2020, whereby countries launched massive mass screening, testing, and containment and mitigation measures [6]. African countries, international health agencies and partners have united and endorsed the implementation of the Joint Continental Strategy. In early February 2020, the Bill & Melinda Gates Foundation committed US$20 million funding to support such response efforts in Africa [7]. According to world bank report 2021, Ethiopia is a country in sub-Sahara Africa with low-income economy [8]. 

The Ethiopian government and Jack Ma Foundation have joined the continental initiatives and distributed medical supplies, including diagnostics and equipment to each of the 55 countries in Africa [9]. 

From 13 March 2020, until 26 July 2022, a total of 491,917, COVID-19 cases with 7568 deaths were identified in Ethiopia [10]. Ethiopia is one of the most populous countries in East Africa, and its government has been implementing various interventions to contain the pandemic [11]. Despite that, during the early phase of the pandemic, it was difficult to determine the real number of COVID-19 cases due to limited availability of COVID-19 testing sites [12].

The Government of Ethiopia established a national COVID-19 response and coordination platforms, and implemented response measures in collaboration with development partners, donors and private sectors [13]. The national preparedness and response coordination platform through an emergency operation center, has been implementing enormous pandemic response measures [14]. However, the pandemic response challenges in Ethiopia have not been properly documented. Besides knowledge on pandemic response challenges, there is limited local evidence on how to make the Ethiopian healthcare system more resilient to pandemics—in the context of this paper, the healthcare system should be able to deal with disruptions, to manage and recover from shocks, bounce back, provide ongoing health services and anticipate future failures [15]. 

This study is aimed at reviewing the COVID-19 pandemic response challenges of the Ethiopian healthcare system. The six WHO building blocks were used as a conceptual framework to describe the pandemic response challenges in Ethiopia.

## 2. Materials and Methods

The Preferred Reporting Items for Systematic Reviews and Meta analyses Statement (PRISMA) [16] was used to report each stage of the literature review and findings.

### 2.1. Search Strategy 

Systematic search was conducted using the electronic databases PubMed andProQuest; manuals, guidelines, and reports from the Ethiopian Federal Ministry of Health were also consulted. Articles focusing on the COVID-19 pandemic in Ethiopia published up to August 2021 and written in English were included. The following key terms ‘pandemic’, ‘challenges’, ‘resilience’, ‘COVID-19’, ‘SAR-COV’, Ethiopia’, ‘Addis Ababa’ were used and combined with Boolean operators ‘OR’ and ‘AND’.

### 2.2. Study Selection

**Figure 1:**
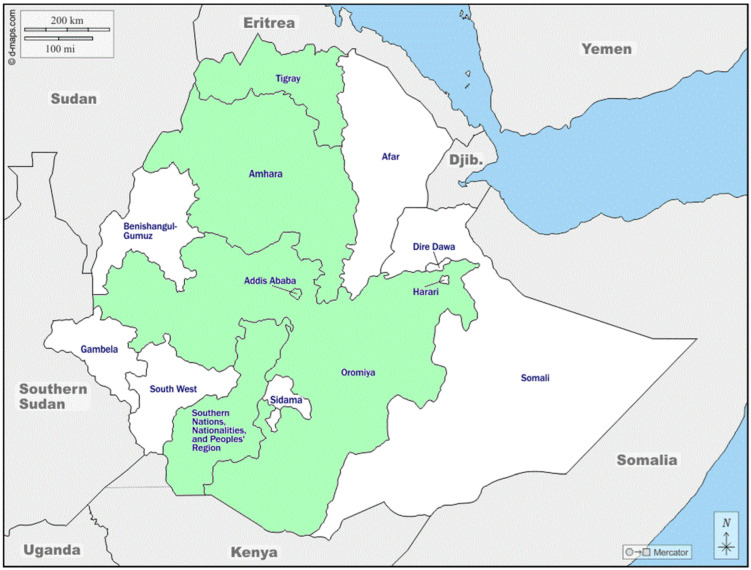
Map of the locations of the studies.

Comprehensive screening of titles and abstracts was performed upon downloading articles from the electronic databases. Studies that did not match the research question along with all the duplicates articles were excluded. Inclusion and exclusion criteria (Table 1) were developed based on the research questions and study locations are reported as follow (Figure 1).

An overview of the studies’ inclusion and exclusion criteria details is reported in Table 1.

### 2.3. Analysis and Reporting the Results

Thematic content analysis was used to analyze the narrative account of the data extracted from the included studies based on the six WHO health system building blocks [17]. Data was extracted around the following themes: Leadership and Governance, Healthcare Financing, Workforce Development, Service Delivery, Essential Medicines, and Information System.

## 3. Results

**Figure 2:**
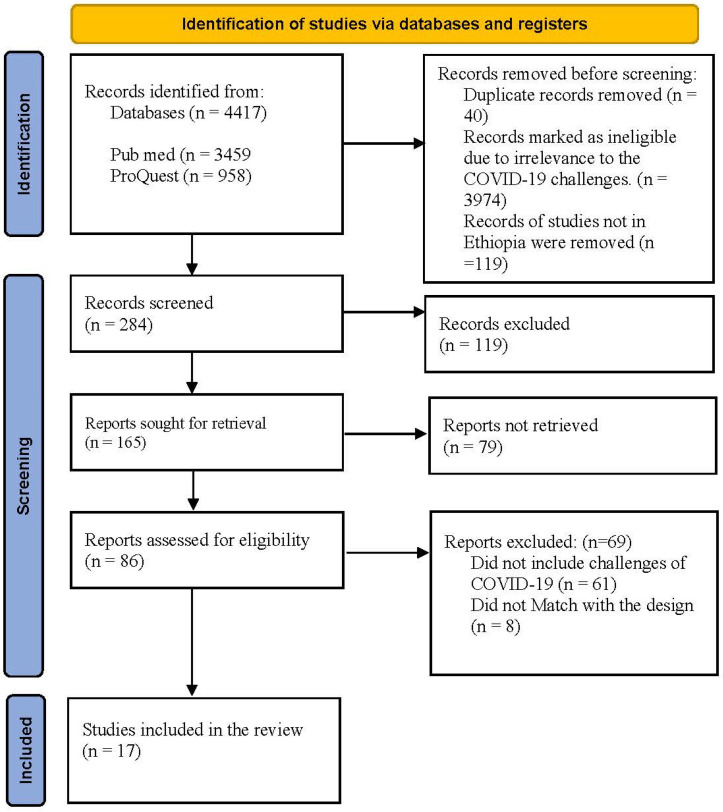
PRISMA model.

A total of 4417 eligible studies were identified through a data bases search (Figure 2). After duplicate removal and screening, 86 studies were retained for eligibility assessment. A total of 69 studies were excluded after abstract screening, thus reducing the articles eligible for full text reading to 17 articles. All 17 articles were then retained in the review (Table 2).

An overview of the studies’ details is reported in Table 2.

### 3.1. COVID-19 Response Challenges to the Healthcare System in Ethiopia

In the following sections, the main challenges identified in the literature regarding the response to the COVID-19 pandemic from the Ethiopian healthcare system are reported following the frame of the six WHO health system building blocks [16].

### 3.2. Leadership and Governance

Strong leadership and governance are considered vital components for the success of any country’s healthcare system and for health system resilience [35]. In Ethiopia, policy makers and sectoral leaders’ support has been vital, as it helped to reduce the COVID-19 spread. For example, policy makers had extensive involvement in the establishment of a national pandemic preparedness and response coordination platform through the Ethiopian public health institute, and emergency operation centers (EOC), where a high-level ministerial committee played a leadership role at the national level [36,37].

With the leadership of the ministerial committee, the government of Ethiopia decentralized COVID-19 care. In particular, the government decided to implement COVID-19 care and laboratory services through the involvement of government and private healthcare facilities, both at national and sub-national level as part of policy initiatives. In addition, to generate evidence for decision making, the country conducted the initial COVID-19 readiness assessments and documented priority gaps and, particularly regarding intensive care services [29]. 

Following a decision of policy makers in Ethiopia, the government did not implement a full-scale pandemic lockdown, but it rather implemented pandemic-preventive measures to reduce the spread of the virus in the country, thus obtaining successful outcomes [36,38]. Nevertheless, during the earlier phase of the pandemic, there were decrease in the visits to the health facilities, and the pandemic resulted in obstacles to the implementation of community-based healthcare services and community health initiatives [27].

### 3.3. Health Financing

With regard to government financing, in Ethiopia government budget allocation to the health sector has increased, reaching 10% in 2020, with more budget allocated to the health sector following the COVID-19 pandemic [39]. In Ekka Kotebe, the largest COVID-19 treatment center in Ethiopia [40], with the commitment of the government, the estimated total cost of COVID-19 treatment was USD 3.75 million with average cost of 1, 473 USD per treated episode [41]. Despite this, the pandemic affected the healthcare system’s financing [19], leading to increased opportunistic cost due to the pandemic and impacting implementation of public health measures in Ethiopia [20]. The financial constraints led to a shortage of essential medical items. For instance, there were shortages of mechanical ventilators, scarce healthcare system capacity to avail full packages of personal protective equipment for health facilities, limited inpatient capacity, as well as isolation and intensive care capacity [32,33].

### 3.4. Health Workforce

The COVID-19 pandemic has burdened the healthcare system, demanding implementation of measures to build a strong healthcare workforce. In Ethiopia, frontline healthcare workers have a critical role in timely and effectively responding to any health crises and enhancing health system performance [39,42]. An effective plan to protect healthcare workers in the working environment is essential in each phase of a pandemic [40,43]. Yet, in Ethiopia, during the COVID-19 pandemic, frontline healthcare workers have been particularly affected by the pandemic and there was shortage of protection measures during the earlier phases of the pandemic, which in turn hampered the coordination and delivering of health services [19]. Also, the laboratory testing capacity was limited due to a shortage of manpower for quality sample collection, transportation, testing and infrastructure, and limited healthcare capacity especially when it comes to the Intensive Care Unit (ICU) [18]. In addition, healthcare workers’ fear of contracting and spreading the virus to their close families and friends, particularly elderly and those who have chronic medical conditions [41,44], was among the main challenges to an effective medical response to COVID-19. This is particularly challenging for Ethiopia, because of its limited trained human resources as compared to the growing healthcare demand [13].

### 3.5. Service Delivery

One of the key functions of the healthcare system in any country is ensuring the availability, ensure access to and delivery of health services that meet minimum quality standards. During the COVID-19 pandemic in Ethiopia there was a shortage of supplies and infection prevention and control measures, which ultimately affected the quality of healthcare services delivery [15,27]. During the earlier phases of the pandemic, the routine essential healthcare services, including maternal health and child and new-born health, was challenged [20,42,45]. As a result, the healthcare system was struggling with the pandemic crises; we found that health care services such as outpatient visits, emergency room visits, inpatient admissions, declined; in addition, there were decreased numbers of elective surgical services, decreased medical, emergency and outpatient flows [21], which predisposed patients to complications. This decline was partially due to decreased demand for healthcare services during the pandemic and lack of the healthcare system resilience. The maternal health service utilization was found to be low, and routine vaccination coverage among children aged 15–23 months remained low and further decreased after the COVID-19 outbreak [22,26,43,46]. The impact of this could have long-term consequences such as failure to pursue the targets set by the Sustainable Development Goals, including a reduction of child and maternal mortality and substantial HIV/AIDS, tuberculosis, and malaria prevention and control measures [28,42,44,45,47].

### 3.6. Essential Medicines

The pandemic has affected the global medical supply chain. Due to this, the government was struggling to address the critical demand for healthcare facilities for essential medicines and personal protective equipment’s (PPE). With respect to essential medicines, Ethiopia has a supportive national policy of prevention and control of public health threats and of institutions responsible for availing essential medicines, including essential medicine lists and treatment guidelines. However, the pandemic has greatly impacted the pharmaceutical supply chain in Ethiopia. For example, during the early phase of the pandemic the healthcare system was unable to forecast and absorb shocks and unable to ensure continuity of essential medicines in the healthcare system. Accordingly, healthcare facilities were facing shortages of medical supplies, ventilators and medical equipment [48]. Shortage of emergency care supplies such as oxygen supply and well-ventilated isolation rooms was reported [20]. In addition, scarce resources for procurement of essential medicines hamper earlier detection, diagnosis, and treatment [20]. Thus, limited testing kits and logistic constraints lead to inadequate testing and tracing capacity at the healthcare facility level.

### 3.7. Information System

Health information systems comprise surveillance reporting, healthcare records and other health databases that help to guide policy response decisions [49]. Public health surveillance provides evidence to understand and predict the pandemic; accordingly, investments in surveillance help to guide actions for the success of the country’s health system [35]. In Ethiopia, after the first case was reported in March 2020 the country started implementing intensive contact tracing, cross-border measures such as mandatory quarantine and surveillance reporting for international arrivals. Apparently, this contact tracing and testing measure has minimized the number of imported cases [34].

## 4. Discussion

This study highlighted the main challenges faced by the Ethiopian healthcare system when responding to the COVID-19 pandemic, paving the way for policy-level recommendations for the health sector that could foster health system resilience in Ethiopia.

The weak healthcare system in most African countries hindered the achievement of effective home and facility based-care of COVID-19 cases, as recommended by WHO [50]. Overall, the healthcare system in Ethiopia was ill-prepared and was not resilient. Care was disrupted for many non-COVID-19-related services. In particular, medical emergency care and surgical services [22], as well as routine maternal and child healthcare services [26], were interrupted. The pandemic continues imposing huge challenges, affecting regular visits to health facilities for both communicable and non-communicable diseases. Guaranteeing continuity of care during disasters is key to avoid a secondary surge of chronic care needs in the impacted population, which has the potential to impact the health system in the months after a disaster leading the health system to collapse [51].

A shortage of ventilators and ICU beds affected the pandemic response in Kenya [52]. Similarly, this review identified a shortage of medicines and medical supplies, such as mechanical ventilators, in Ethiopia. This shortage affected the delivery of healthcare services through limiting inpatient services, isolation, treatment, and intensive care capacities in a moment of increased healthcare demands. Nevertheless, private enterprises have mobilized in support of the Ethiopian government, contributing to the financing of activities to combat the spread of the virus, including the increase of the testing capacity, the creation of an isolation center, and the supply of personal protective equipment for medical personnel [53].

Strong leadership and governance is paramount for the success of the healthcare system [35]. In Ethiopia, at the policy level, the prime minister’s office, ministerial engagement and leadership played a vital role in the pandemic response. The government commitment was strong to address the essential medicine shortage, imposing legal measures to interrupt the spread of the virus. The senior leadership of the country mobilized resources from multiple sources and allocated them for the procurement of essential medicines, implementing non-pharmaceutical measures, and striving to enhance the intensive care unit and the laboratory capacity [18,20]. The implementation of strict measures, such as lockdown, was not feasible in many low-income countries [54]. Accordingly, the government of Ethiopia implemented preventive measures such as massive thermal screening, rapid identification of suspects, contact tracing, isolation, quarantine, and treatment of infected cases as a vital strategy with successful outcomes. This strategy proved to be good for a country with a fragile health system, already compounded by conflicts, outbreaks and inflation. This shows the importance to tailor a disaster response strategy to the local context, considering not only the impact of the disaster event itself, but also the possible snowballing consequences of containment measures and infection prevention and control interventions.

With regard to healthcare financing, in many African countries government expenditure for the health sector was relatively low [55] and the pandemic further affected healthcare system financing capacity [19]. Following the pandemic, leaders and policymakers in Ethiopia allocated scarce resources to address the massive needs of the health sector and, consequently, government and donor’s expenditure for the pandemic response increased. The government assigned financial resources for renovation of care facilities, expansion of care centers, procurement of supplies and incentives for healthcare workers. Despite government efforts to strengthen the capacity of the healthcare system and financial allocation commitment for the government, healthcare facilities remain ill-prepared for pandemics particularly due to financial and logistic constraints. This requires sustainable pandemic healthcare financing for better pandemic preparedness, response, continuity of care and healthcare resilience.

Overall, this review shows that multi-sectoral collaboration including private sectors, not-for-profit and faith-based organizations is likely to play a vital role in building healthcare resilience and ensuring provision of uninterrupted essential healthcare services. This collaboration requires the understanding of how the public and private health sectors interact during shocks [55]. The pandemic response in Ethiopia has implemented the response measures through coordination and collaboration with multi-sectoral agencies including government sectors, donors, development partners, and private institutions, with the leadership of the health sector. Nevertheless, the role of the private and non-governmental development partners in pandemic preparedness, response and resilience building should be better delineated and formalized. Decentralized leadership and strong healthcare facilities’ governance are necessary to build a resilient healthcare system.

Creating a resilient health system is one of the key priorities in Africa. In 2016, the WHO endorsed the International Health Regulations (IHR) Joint External Evaluation (JEE) tool designed to measure countries’ capacities to execute and ability to prevent, detect, and respond to public health emergencies of international concern as per the recommendations of the IHR. Accordingly, the country-specific focus should be conducting regular assessment of country preparedness capacity according to the IHR-JEE tools and implementing corrective measures to build pandemic preparedness and resilience capacity [1,56]. To do this, there is a recognized need to generate evidence, build the capacity of the healthcare system and enhance healthcare system resilience in Ethiopia.

### 4.1. Key Messages

(a) The Ethiopian pandemic response saw good support from policymakers and Ministry of Health authorities;
(b) No large-scale lockdown was enforced in Ethiopia, but preventive measures were privileged;
(c) Shortage of medical personnel resulted in scarce testing capacity;
(d) Ethiopia experienced disruption of routine health services, including MNCH services;
(e) The pandemic impacted the pharmaceutical supply chain, leading to a shortage of emergency care supplies;
(f) The private sector had an important role in supporting the health system in the response to the COVID-19 pandemic in Ethiopia.

### 4.2. Limitations and Strengths

This review is not without limitations. Only two scientific databases were screened, and no grey literature was consulted. The lack of consultation of the EMBASE database is a limitation of this study, but no institutional access was granted for it. Given the wide overlap with MEDLINE, the authors are confident that no relevant study was missed. Some strengths of this study are also pointed out. First, this review followed a systematic process for data retrieval and screening. Second, the primary analysis of records was performed by a researcher currently living and working in Ethiopia, thus ensuring deep knowledge of the context and the Ethiopian pandemic management process. Third, the reliance on a well-known framework, namely the six WHO healthcare system building blocks, allows the study to be understood by policymakers and the broader public, as well as it to be compared with similar studies in other contexts. Fourth, the recommendations and key messages elaborated in this study have been tailored to the specific context of Ethiopia, facilitating the translation of knowledge into policy practice.

## 5. Conclusions

Ethiopia has implemented an abundant pandemic prevention and control measure, with the active participation of policy makers’ leadership. Following the pandemic, healthcare facilities were facing shortages of emergency care medical supplies, ventilators, medical equipment, oxygen supply and well-ventilated isolation rooms. This review shows that strong preparedness and multi-sectoral collaboration play a vital role in building resilience and continued delivery of essential healthcare services. Our finding identified that, in Ethiopia, building healthcare resilience is key and should be taken into account for future pandemic responses to maintain continuity of healthcare services. We recommend policymakers and healthcare system representatives to enhance pandemic preparedness and response capacities by targeting the six WHO health system building blocks. Further studies are warranted on the role of private and non-governmental organizations on pandemic response, resilient healthcare system, and policy level perceptions and barriers for building disaster resilient healthcare system.

## Figures and Tables

**Table 1:** Inclusion and exclusion criteria.

Inclusion Criteria	Exclusion Criteria
(1) The study deals with the health response and management of the COVID-19 pandemic.	(1) The study is conducted in other countries than Ethiopia.
(2) The study setting is Ethiopia.	(2) The article is written in other languages than English.
(3) The article is written in English.	(3) The article’s full text is not available.

**Table 2:** Details of studies included in the review.

Authors/Ref	Publication Date	Methodology Used	Study Aim	Population and Sample Size	Identified Challenges to Pandemic Response
Tulu et al. 2020 [18]	9 July 2020	Description of events	To describe the general epidemic preparedness of Ethiopia.	NA	Limited COVID-19 care capacity. Inadequate intensive care units (ICU).
Mulu, et al. 2020 [19]	16 September 2021	Facility-based cross-sectional	To assess the preparedness and responses of healthcare providers to combat the spread of COVID-19 among North Shewa Zone Hospitals, Amhara, Ethiopia.	422 healthcare providers	Increased care demands. Inadequately prepared healthcare system. Limited inpatient admission capacity.
Shimeles et al. 2021 [20]	8 July 2021	multi-facility-based cross-sectional	To assess the trend of health service utilization and challenges faced during the COVID-19 pandemic at primary units in Addis Ababa, Ethiopia.	NA	Insubstantial facility infrastructure. Decline in the patient flow. Disparity in service delivery. Shortage of medical supplies.
Mersha et al., 2021 [21]	22 January 2021	semi-structured interview	To explore barriers affecting the practice of preventive measures for the COVID-19 among health professionals.	16 key informants	Unavailability of guidelines. Water supply shortage. Shortage of emergency rooms and problems with triage.
Abdela et al., 2020 [22]	5 August 2020	Description of events	To understand the reasons behind the decrease in patient flow by referral Hospitals in Ethiopia.	NA	Chronic disease follow-up decreased. Visits at the emergency service decreased. Both neonatal and other childhood emergency visits decreased.
Shimeles et al., 2021 [23]	6 April 2021	Multi-site cross-sectional	To assess the magnitude and associated factors of poor medication adherence among diabetic and hypertensive patients visiting public health facilities in Addis Ababa, Ethiopia during the COVID-19 pandemic.	422 participants	Poor adherence to anti-diabetic and, antihypertensive medications.
Shigute et al., 2021 [24]	19 May 2020	Description of events	To describe the balance in access during the pandemic.	NA	Some public hospitals reduced routine care and have been providing care exclusively for COVID-19 patients. Out-patient and in-patient services interrupted.
Abraha et al., 2021 [25]	10 March 2021	Secondary data	To describe the clinical features and assess the determinants of severity and in-hospital mortality of patients with coronavirus disease 2019 (COVID-19) in a unique setting in Ethiopia.	2617 patients admitted to COVID-19 center	Increased NCDs comorbidities such as cardiovascular diseases, including hypertension, diabetes and chronic obstructive lung diseases, including asthma. Co-infection with HIV as risk factor for developing severe COVID-19.
Miretu et al., 2021 [26]	20 March 2021	Community-based cross-sectional study	To assess the impact of COVID-19 on vaccination coverage among children aged 15–23.	633 children with their mother/caregiver were interviewed	Decreased maternal health service utilization. Decreased full vaccination coverage following the COVID-19 pandemic.
Birihane et al., 2020 [27]	20 November 2020	Primary data	To assess perceived barriers and preventive measures of corona virus disease among healthcare providers in Debretabor Town, north central Ethiopia.	healthcare professional *n* = 203.	Insufficient training for healthcare workers. Inadequate adherence to precaution measures.
Mekonen et al., 2020 [28]	5 January 2021	Primary data	To assess the prevalence and associated factors of anxiety, depression, and stress among nurses working in northwest Amhara referral hospitals.	302 nurses	Increased prevalence of anxiety. Depression and, stress among nurses. Fear of infecting family members.
Mohammed et al., 2020 [29]	16 September 2020	Description of events	To describe the COVID-19 containment measures and its implication on tuberculosis care and research in Ethiopia.	NA	Pandemic affected the healthcare system functions. Diagnosis, care, and treatment, human and material resources for TB have been shifted to COVID19. Weak TB patients transferred to other health facilities.
Wodajo et al., 2020 [30]	8 March 2020	Record review	To assesses the impact of the COVID-19 response on the TB control activities of Addis Ababa health centers.	56 selected public health clinics	Flow of patients markedly decreased during the COVID-period.
Seid et al., 2020 [31]	27 August 2020	Description of events	Describing COVID-19 impact on NTD prevention and care.	NA	The pandemic postponed mass campaigns and validation surveys. Irregular community program supportive visit. Working guidelines not timely distributed to health facilities.
Tiruneh et al., 2021 [32]	10 February 2021	Institutionally based survey	To assess the level of hospital preparedness for COVID-19 in South Gondar Zone Governmental Hospitals, 2020.	NA	Shortage of mechanical ventilators. Limited (ICU) equipment’s and treatment capacity.
Boti et al., 2021 [33]	19 March 2021	Phenomenological	To explore the barriers to effective implementation of public health measures for prevention and control of the COVID-19 pandemic in the Gamo Zone of southern Ethiopia.	NA	Financial constraints lead to shortage of PPE.
Gudina et al., 2020 [34]	29 March 2021	Surveillance data base	To describe the epidemiology of COVID-19 in Oromia Region, the largest and most populous region in Ethiopia, during the early months of the outbreak.	NA	Surveillance data was not sufficiently used to identify group and/or area most exposed.

NA: Refered as Not Applicable.

## Data Availability

All the data used for this study is available within the manuscript.
